# Treatment fidelity of brief motivational interviewing and health education in a randomized clinical trial to promote dental attendance of low-income mothers and children: Community-Based Intergenerational Oral Health Study “Baby Smiles”

**DOI:** 10.1186/1472-6831-14-15

**Published:** 2014-02-24

**Authors:** Philip Weinstein, Peter Milgrom, Christine A Riedy, Lloyd A Mancl, Gayle Garson, Colleen E Huebner, Darlene Smolen, Marilynn Sutherland, Ann Nykamp

**Affiliations:** 1 Northwest Center to Reduce Oral Health Disparities, Department of Oral Health Sciences, Box 357475, University of Washington, Seattle, WA 98195-7475, USA; 2 Klamath County Public Health, 403 Pine Street, Klamath Falls 97601, OR, USA

**Keywords:** Dental health, Motivational interviewing, Clinical competence, Postpartum care, Prenatal care

## Abstract

**Background:**

Fidelity assessments are integral to intervention research but few published trials report these processes in detail. We included plans for fidelity monitoring in the design of a community-based intervention trial.

**Methods:**

The study design was a randomized clinical trial of an intervention provided to low-income women to increase utilization of dental care during pregnancy (mother) or the postpartum (child) period. Group assignment followed a 2 × 2 factorial design in which participants were randomly assigned to receive either brief Motivational Interviewing (MI) or Health Education (HE) during pregnancy (prenatal) and then randomly reassigned to one of these groups for the postpartum intervention. The study setting was four county health departments in rural Oregon State, USA. Counseling was standardized using a step-by-step manual. Counselors were trained to criteria prior to delivering the intervention and fidelity monitoring continued throughout the implementation period based on audio recordings of counselor-participant sessions. The Yale Adherence and Competence Scale (YACS), modified for this study, was used to code the audio recordings of the counselors’ delivery of both the MI and HE interventions. Using Interclass Correlation Coefficients totaling the occurrences of specific MI counseling behaviors, ICC for prenatal was .93, for postpartum the ICC was .75. Participants provided a second source of fidelity data. As a second source of fidelity data, the participants completed the Feedback Questionnaire that included ratings of their satisfaction with the counselors at the completion of the prenatal and post-partum interventions.

**Results:**

Coding indicated counselor adherence to MI protocol and variation among counselors in the use of MI skills in the MI condition. Almost no MI behaviors were found in the HE condition. Differences in the length of time to deliver intervention were found; as expected, the HE intervention took less time. There were no differences between the overall participants’ satisfaction ratings of the HE and MI sessions by individual counselor or overall (p > .05).

**Conclusions:**

Trial design, protocol specification, training, and continuous supervision led to a high degree of treatment fidelity for the counseling interventions in this randomized clinical trial and will increase confidence in the interpretation of the trial findings.

**Trial registration:**

ClinicalTrials.gov: NCT01120041

## Background

Patient-centered care is now promoted in medicine. Evidence that it has taken root is presented in the Institute of Medicine report that includes patient-centered care as one of the six domains of quality [1]. The Commonwealth Fund 2003 Survey of Physicians and Quality of Care found that one-fourth of primary care physicians incorporate patient-centered approaches in their practices [2]. Motivational Interviewing (MI), a counseling technique found to be highly effective with addiction disorders [3], has been adapted to achieve various health-enhancing objectives (e.g., dietary change) [4]. While the health goals, training and the professional role of the interventionists, number of patient contacts, and duration of sessions may vary, the choice to use MI techniques reflects a common value of patient-centeredness.

In the oral health field there have been at least 40 studies that utilized patient-centered approaches--typically an abbreviated form of MI--to deliver behavioral interventions to enhance patients’ oral health. Reviewers have cited MI as the most promising intervention for the control of Early Childhood Caries [5-7]. However, attributing intervention success to MI per se requires more information about its implementation and specifically how extensively and how well the MI techniques were used.

Treatment *fidelity* refers to assessment and monitoring of an intervention as it is actually delivered to patients, clients or study participants. Assessment of fidelity is key to the validity of any behavioral intervention study. Borrelli [8] notes that treatment fidelity increases confidence that changes in the primary outcome of a trial are the result of the experimental treatment and not other factors. Treatment fidelity has two components: 1) treatment integrity, which is the degree to which the treatment is delivered as intended by the researchers; and 2) treatment differentiation, the extent to which the intervention and comparison differ on dimensions assumed to influence outcomes [9-12].

Within the oral health behavioral research literature, reports of treatment fidelity are rare: only one published study has reported such assessments [13]. The study of a behavioral intervention to control Early Childhood Caries among African-American preschool children used MI techniques to help caregivers achieve their own prevention goals. The training and fidelity of the MI interventionists was reported. The authors provided details of how the interventionists were trained and their adherence to the study protocol over the period of the intervention. They found there was weak fidelity to the intervention and limited effectiveness.

The purpose of this study is to describe the design, training, and methods of ongoing fidelity monitoring in the Community-Based Intergenerational Oral Health Intervention Study “Baby Smiles”. The primary objectives of the intervention are to increase utilization of dental care by low-income women during their pregnancy, as well as to increase utilization of preventive dental care by their children by 18 months of age. The rationale is that dental treatment during pregnancy, and age one preventive visits, contribute to both improved pregnancy outcomes and lower incidence of Early Childhood Caries [14,15].

## Methods

### Study design

Our test of the intervention used a 2 × 2 factorial design in which participants were randomly assigned to one of four treatment arms: brief Motivational Interviewing (MI) during pregnancy (prenatal) or postpartum or both or Health Education (HE). The study design is shown schematically in Table 1.

**Table 1:** Study design of the Community-Based Intergenerational Oral Health Intervention Study “Baby Smiles”

		**Child (Postpartum) MI**	
		**Yes**	**No**
**Pregnancy (Prenatal) MI**	**Yes**	Group 1	Group 2
		Prenatal MI—Postpartum MI	Prenatal MI–Postpartum HE
		N=145	N=59
	**No**	Group 3	Group 4
		Prenatal HE –Postpartum MI	Prenatal HE –Postpartum HE
		N=146	N=50

### Study setting

The study was conducted in four rural counties (Douglas, Lincoln, Jefferson, and Josephine) in Oregon State USA. The intervention was delivered in a Women, Infants, and Children Center (WIC) or a public health department. WIC is a USA federal government program to ensure proper nutrition for low income mothers and their children.

### Study participants

Participants were 400 English-speaking women and their live-born children living in the four rural Oregon counties. To be eligible, the women had to be at least 15 years of age, in their first or second trimester of pregnancy, and eligible for coverage in the Oregon Health Plan Plus, which provides medical and dental services to adults and children enrolled in Medicaid. Medicaid is a state-administered national government system of health insurance for those requiring financial assistance. The Institutional Review Board of the University of Washington and the Public Health Institutional Review Board of Oregon state approved the study.

### Assignment to study conditions

Participants were randomly assigned to one of the four intervention groups using computer-generated permuted blocks of varying block sizes to ensure that the groups were proportionally balanced across study period and within each county and counselor. The randomization procedure was stratified on county and counselor.

### Study interventions

The interventions utilized either brief Motivational Interviewing (MI) or traditional Health Education (HE) to provide oral health education, assist women to adopt behaviors associated with optimal oral health, and to seek professional dental care for themselves and their young children. Five counselors were chosen by the local health departments (there were two counselors in one county because enrollment took place in two locations) to be trained and deliver both interventions. In the brief MI conditions, counselors utilized typical MI techniques including open-ended questions, reflective listening, and affirmations [16]. The purpose was to create a discussion that engaged the participant in thinking about, and planning how to make, positive behavioral changes. The goal of the HE intervention was to improve oral health-related behavior also, but not through a patient-centered dialogue. In the HE condition, counselors played videos, stopped the videos at prescribed points to ask, “Do you have any questions?” and provided the participant with print materials from the National Maternal Child Oral Health Resource Center at Georgetown University (http://www.mchoralhealth.org). Participants in both intervention groups received printed handouts about how to use their dental care insurance coverage, what to expect at a dental visit and other written recommendations developed for the study. All the materials and the full study protocol are available on the website of the Northwest Center to Reduce Oral Health Disparities (URL: http://depts.washington.edu/nacrohd/babysmiles). Additional detail about each intervention, as it was delivered in the prenatal and postpartum periods, is provided below.

#### MI prenatal

When the participants were assigned to the prenatal MI treatment arm (Table 1), participants received individual in-person counseling. The counselor attempted to establish a therapeutic alliance, identify and reinforce dental needs, assess and share dental risks, and identify and help navigate barriers to care. During the single session, the counselor utilized both a written and computer-guided protocol to deliver the intervention and show, based on the counselor’s assessment of a participant’s need, a maximum of five very brief videos on key points (*e.g*., “Baby teeth are important because if there is an infection in the baby teeth, there will be an infection in the permanent teeth…”). At the conclusion of the intervention, the counselor offered to assist making an appointment with a dentist serving Oregon Health Plan clients. Within six weeks of the in-person session, the counselor made up to two follow-up telephone calls to provide support, to identify problems, and problem solve. Additionally, participants received a postcard one month prior to scheduled baby’s birth. Its purpose was to inquire about their pregnancy as well as address dental concerns. There was also a phone contact one week after the due date to check in, get the child’s Medicaid Identification Number if known, confirm the mailing address for the baby gift, and schedule the three month postpartum visit when possible. There was a second call at 6 months post partum, which was used to check in with the mother and to maintain contact with our study participants.

#### MI postpartum

When the participants were assigned to the postpartum MI treatment (Table 1), they attended a MI session approximately nine months after their baby was born. As in the prenatal MI intervention, establishing a therapeutic alliance, identifying needs and problem solving were counselor goals. Potential behavioral goals for the mother were presented as an Early Childhood Caries prevention menu with information about oral hygiene and dietary practices, and the age one dental visit. Mothers identified menu items they were interested in and barriers to implementation were identified and discussed. The mother was offered assistance in making an appointment for the child with a dentist serving Oregon Health Plan clients. This session was followed by one telephone call about six weeks afterwards to identify problems with the achievement of the mother’s stated goals.

#### HE prenatal

When participants were assigned to the prenatal HE treatment arm (Table 1), each received a traditional health education intervention. The health information included a 15 minute video and the pamphlet “Two Healthy Smiles” available from the National Maternal Child Oral Health Resource Center at Georgetown University (http://www.mchoralhealth.org). Topics presented included maternal dental health and transmission of decay causing bacteria from mother to child.

#### HE postpartum

In the postpartum HE condition (Table 1), the materials included a ten-minute video about how to prevent Early Childhood Caries and two pamphlets “Your Young Child” and “Topical Fluoride Recommendations For High Risk Children” from the National Maternal Child Oral Health Resource Center at Georgetown University (http://www.mchoralhealth.org). Topics of the video included the importance of baby teeth, dietary and hygiene recommendations, and baby’s first dental visit. In both HE interventions participants were offered assistance in making dental appointments. To maximize participant attention and community acceptance, the videos were relatively brief. The length of all sessions was recorded.

### Treatment fidelity measures

Our approach to fidelity monitoring was based on the framework of Bellg and colleagues [17] that focused on three elements: (1) study design, (2) training interventionists, and (3) delivery, receipt and enactment of treatment skills during the intervention.

### Study design

Two major activities were embedded in the study design to ensure fidelity of the intervention. A standardized protocol manual–a detailed written, fill-in the blank protocol with an accompanying guide—was developed for the counselors, and the type and number of contacts per participant within the MI and HE treatment arms was standardized.

### Counselor training

There were four major activities to ensure the counselors developed the skills to deliver the intervention with high fidelity and would maintain fidelity over time: (1) standardized training, (2) assurance of skill acquisition, (3) minimization of drift in skills, and (4) accommodating provider differences. The counselors were trained in MI techniques and in the protocol by one of the investigators (PW). Initial training was provided in a 10-hour in-person session. Then each counselor, based on perceived need, received additional sessions of one to two hours of personalized instruction by telephone and videoconference. Exercises focusing on reflective listening, providing affirmations, and identifying and exploring resistance were included. Role-playing with the protocol utilizing sample participant scenarios was included in every session. Counselors were certified before being allowed to deliver MI or HE to study participants. Audio recordings were made of telephone role plays for each of the counselors. Counselors were certified if their MI skills during the role play were scored as adequate, very good or excellent (competence rating: (1) Very poor; (2) Poor; (3) Adequate; (4) Very Good; and (5) Excellent). Two “booster” one-hour group videoconferences were provided post certification. In addition, counselors received brief (15 minute to half hour) personalized telephone feedback based on audio recordings of their sessions.

Near the end of the study, three trained counselors were replaced by a single full-time employee (no counselor was replaced for poor counseling performance). Her training included viewing a video made of the initial training session, along with the same training and certification process completed by the initial set of counselors. Every week a “MI tip of the week” was provided to counselors at a weekly conference call to reinforce training and minimize drift. Prior to the initiation of the postpartum phase, counselors received an eight hour in-person training session, certification exercises, and two post-certification booster video conferences. Counselors also received brief telephone feedback from the audio recordings that were coded.

### Coding

All sessions were audio recorded. Twenty percent of the recordings were utilized to assess intervention delivery. Recordings were coded by a research team member who is the gold standard (PW), and other coders who were trained by PW. The coding scheme was based on a modified Yale Adherence and Competence Scale (YACS)--a system of 15 constructs each rated on two seven-point scales--developed to rate adherence and competence in providing behavioral treatments for substance use disorders. YACS scales are reliable and have been shown to have construct and discriminant validity [18].

We identified 10 constructs from YACS that were congruent with our MI intervention. Eight constructs were “MI consistent”: (1) MI Style or Spirit, (2) Fostering a Collaborative Relationship, (3) Providing Statements of Support/Affirmations, (4) Reinforcing Motivation to Change/Encouraging Change Talk, (5) Use of Open-Ended Questions, (6) Recognizing and Exploring Resistance, (6) Developing Discrepancy, and (8) Reflective Listening. Two constructs reflected behaviors that should be minimized in an MI counseling approach (MI Inconsistent): (1) Unsolicited Advice, Directions, and (2) Feedback and Direct Confrontation. Each of these constructs was rated for adherence and competence using a five-point Likert scale with the following response categories: 1 = Very Poor, 2 = Poor, 3 = Adequate, 4 = Very Good, 5 = Excellent corresponding to the YACS anchors of 1 = Not at all to 5 = Extensively.

### Coding fidelity

One investigator (PW), who functioned as the gold standard, trained the coders. A Fidelity Coding Manual was written to operationalize each rating scale and to minimize bias. A copy of the guide is on the website of the Northwest Center to Reduce Oral Health Disparities (URL: http://depts.washington.edu/nacrohd/babysmiles). During the training, and afterwards, coder meeting notes were kept and were the basis for modifications to the Fidelity Coding Manual. Small group discussion and recoding was necessary in order to achieve adequate reliability. Because the vast majority of ratings were “adequate” and agreement between raters for “very good” and “excellent” was low, a decision was made to study the frequency of specific MI counseling behavior occurrences regardless of the ratings for each session. Constructs included in the scales were: support, fostering a collaborative relationship, discrepancy, change, open-ended questions, reflective listening, and exploring resistance.

The MI style/spirit was not included in the scales because it was not assessed for HE sessions. We do not report results for advice giving and confrontation because these were behaviors to be avoided and thus the frequency was quite low.

After recoding, interclass correlation coefficients (ICC) were generated between the gold standard coder and each of the two coders based on 14 prenatal and 20 postpartum sessions combined. Because one of the coders left the position before doing a meaningful amount of coding, the reliability coefficients were calculated for a single coder only. Totaling the occurrences of specific MI counseling behaviors described above, the ICC for prenatal sessions was 0.93 (95% CI .81, .98), and for postpartum sessions the ICC was .75 (95% CI .48, .89).

### Satisfaction

Three months after the birth of their child participants completed the Feedback Questionnaire that included six items about satisfaction with elements of the intervention. The items assessed the media components, counselor concern, encouragement in going to the dentist, helpfulness in going to the dentist, ease of talking, and trust. Each was rated on a five-point Likert scale that ranged from zero (Strongly Disagree) to four (Strongly Agree). Eighteen months after the birth of their children, participants completed a similar questionnaire that asked for feedback on the postpartum intervention. Two-way analysis of variance using heteroscedasticity-consistent standard errors to account for unequal variances was used to examine the differences between treatment groups among counselors and the interactions between treatment group and counselor [19].

## Results and discussion

### Treatment differentiation

**Figure 1:**
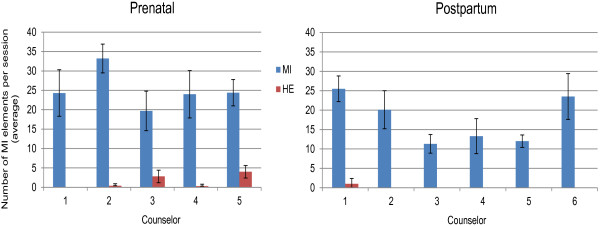
**Average number of specific MI counseling behaviors per session.** Error bars are ± 1 SD.

As shown in Table 2 and Figure 1, all interventionists engaged in all specific MI counseling behaviors at all appointments. Although each counselor delivered both MI and HE interventions, the frequency of MI occurrences in her delivery of the HE condition was very low compared to the frequencies in the MI condition. The competence of MI delivery did not differ among interventionists, while the frequency of MI behaviors varied.

**Table 2:** Treatment fidelity during prenatal and postpartum counseling sessions in the Community-Based Intergenerational Oral Health Intervention Study “Baby Smiles”

		**Number of specific MI counseling behaviors used during session**						**Average MI competence rating (range = 1-5)**		
		**MI participants**			**HE participants**			**MI participants**		
**Study phase**	**Counselor #**	**n**	**Mean (SD)**	**Min - Max**	**n**	**Mean (SD)**	**Min - Max**	**n**	**Mean (SD)**	**Min - Max**
**Prenatal**	**1**	6	24.3 (6.0)	16 - 33	4	0.0 (−)	0 - 0	6	3.0 (−)	3.0 - 3.0
	**2**	5	33.2 (3.7)	27 - 36	5	0.4 (0.5)	0 - 1	5	3.0 (−)	3.0 - 3.0
	**3**	13	19.7 (5.1)	9 - 30	8	2.8 (1.6)	0 - 5	13	3.0 (0.04)	3.0 - 3.1
	**4**	10	24.0 (6.1)	14 - 32	10	0.3 (0.5)	0 - 1	10	3.1 (0.1)	3.0 - 3.2
	**5**	10	24.4 (3.4)	16 - 28	10	4.0 (1.6)	1 - 7	10	3.0 (0.1)	2.9 - 3.1
**Postpartum**	**1**	13	25.5 (3.3)	19 - 29	7	1.0 (1.4)	0 - 4	13	3.0 (0.05)	3.0 - 3.2
	**2**	9	20.1 (4.9)	12 - 26	4	0.0 (−)	0 - 0	9	3.0 (0.1)	2.7 - 3.2
	**3**	12	11.3 (2.4)	6 - 15	8	0.4 (0.7)	0 - 2	12	3.0 (0.1)	3.0 - 3.2
	**4**	16	13.3 (4.5)	6 - 23	5	0.2 (0.4)	0 - 1	16	3.0 (0.1)	3.0 - 3.2
	**5**	5	12.0 (1.6)	10 - 14	1	0.0 (−)	0 - 0	5	3.0 (−)	3.0 - 3.0
	**6**	19	23.5 (5.9)	13 - 33	7	0.1 (0.4)	0 - 1	19	3.1 (0.1)	3.0 - 3.2

MI = Motivational interviewing.

HE = Health education.

When viewing specific MI counseling behaviors such as providing affirmations, open-ended questions, reflective listening, and discrepancy developing questions, and change talk, all counselors had much higher frequencies of these counseling behaviors in the MI than in the HE conditions. Advice giving (unsolicited advice) and confrontation were also coded but there were no instances of confrontation in either the prenatal or postpartum phases and advice giving was limited to less than one advice giving episode per session for both phases.

While a detailed written protocol was provided for the interventions, variation in how the protocol was used was permitted as long as all topics were covered. For example, the menu of choices presented to a participant in the postpartum MI condition could be explored in one pass—identifying possible choices and immediately exploring potential problems, or in two passes, first identifying all possible choices and then identifying problems in the final selection. It may be that the coding of exploring resistance may be a function of the variation chosen, with less resistance identified in the later variation. Differences in the frequency of interventionist behaviors were consistent with our knowledge of the individual styles of the interventionists (e.g., a few open-ended questions to establish rapport, or multiple questions).

### Treatment integrity

#### Study design

That this study is community-based--a partnership between university-based “experts” and rural communities--has implications. With the exception of one replacement counselor, the researchers did not select counselors. Job descriptions and counselor characteristics were provided by the research team and then the local health departments initiated searches for counselors. All five counselors selected by the health departments were already employed in unionized positions. Other studies in community settings faced similar problems [13,20]. Given the lack of opportunity to assess and choose counselors, both the protocol and the training itself were extremely important.

A detailed step-by-step intervention protocol and guide for the use of the protocol was written. The counselors had to learn the protocol, and the written protocol, with space for the counselors to fill in participant responses, was used in every counseling session. Counselors were given some flexibility. They were to word the questions and directions to suit themselves and were allowed to alter the sequence of sections for some participants. However, they were not to omit any section or add any additional material. To minimize variation among counselors, brief video vignettes providing key messages were utilized as part of the intervention protocol.

#### Training

Both group and individual training sessions were provided. Because of the distances involved and the cost of travel, individual training occurred by telephone and video conference. While face-to-face individualized training is always preferable, the phone training--exercises and role plays--were effective as all counselors met basic mastery criteria and were certified.

#### During the intervention

While the counselors adhered to the MI protocol and utilized specific MI counseling behaviors, competence ratings hovered around the midpoint of the competence scale (3 on a 5 point scale). Competence ratings to hover around the midpoint (adequate) by design. The counselors were instructed to deliver the intervention in a consistent manner to each participant with equal enthusiasm.

The MI training sessions emphasized the patient-centeredness of the intervention and prohibited unsolicited advice. Moreover, counselors were trained to recognize resistance and how to circumvent potential confrontations. As a result there was very little advice given and there was no confrontation behavior in either the MI or HE conditions.

Given that counselors provided both MI and HE, it is reasonable to assess possible carryover from one condition to the other. While counselors provided almost no MI behaviors in the HE condition, the lack of advice and confrontation in the HE condition may reflect carryover from MI to HE that could minimize differences obtained from the two interventions.

#### Duration of the intervention

Mean counselor duration for the delivery of the prenatal MI condition ranged from 22 to 34 minutes. Mean duration for the prenatal HE condition ranged from 11 to 19 minutes. On average, MI sessions lasted 11 minutes longer than HE sessions (p < .0001; 95% CI 9.2, 13.2 minutes). In the postpartum sessions, the duration of the MI condition ranged from 17 to 29 minutes whereas the HE condition ranged from 11 to 18 minutes. On average, postpartum MI sessions lasted about 6 minutes longer than HE sessions (p < .0001; 95% CI 5.1, 7.8 minutes).

The number of in-person and telephone contacts in the HE and MI conditions were identical by design. The duration of the two conditions differed and was a function of the length of the videos used in the HE conditions primarily. The videos were relatively brief and presented typical information and encouragement provided to pregnant women and new mothers. This gave us a “real world” comparison. The mothers had the choice to extend the HE sessions with questions and discussion but few did. The differences in duration between the two conditions were not reflected in mothers’ satisfaction ratings.

### Satisfaction

The satisfaction scores are given in the Feedback Questionnaire in Table 3. There were no differences between the ratings of HE and MI participants overall or by individual counselor (p > .05). Average feedback varied by counselor (p = .001), and although the differences were statistically significant for counselors 1 vs. 2 (.4) and counselors 1 vs. 3 (.5), the magnitude of the difference was clinically negligible. Similarly, no differences were found between the ratings of HE and MI participants overall or by individual counselor (p > .05) for the postpartum period. Average feedback did not vary significantly by counselor (p = .81).

**Table 3:** Summary of feedback questionnaire data by intervention phase

	**n**	**Mean (SD)**	**Range**
**Prenatal feedback**			
Video gave useful information	274	3.4 (5.6)	2 - 4
The counselor was concerned about me	275	3.2 (1.8)	0 - 4
The counselor helped me go to the dentist	278	3.4 (3.9)	0 - 4
The counselor encouraged me to go to the dentist	280	3.7 (4.5)	0 - 4
The counselor was easy to talk with	277	3.8 (4.5)	0 - 4
I could trust the counselor	277	3.7 (7.6)	0 - 4
**Postpartum feedback**			
The video I watched gave me useful information	196	3.5 (5.7)	0 - 4
The informational materials gave me useful information	212	3.6 (5.7)	0 - 4
The counselor really cared about me and my child	216	3.8 (1.5)	0 - 4
The counselor helped me find a way to take my child to the dentist	208	3.6 (4.7)	0 - 4
The counselor encouraged me to take my child to the dentist	214	3.8 (5.5)	0 - 4
The counselor was easy to talk with	216	3.8 (6.5)	0 - 4
I could trust the counselor	216	3.8 (2.6)	0 - 4

Lack of differences in participants’ feedback about the HE and MI conditions, in both prenatal and postpartum phases is puzzling. We expected greater satisfaction with the MI intervention: this was not found. Perhaps because the same counselors provided both MI and HE, differences were minimal. On the other hand, these findings may be viewed as indicating a minimum of non-specific treatment effects.

## Conclusions

It is critical to the future interpretation of the main outcomes of the trial that appropriate levels of fidelity were established. Counselors were certified as having met training criteria prior to initiation of this randomized controlled trial. Performance assessment found no overlap in the behaviors of the counselors in MI and HE conditions. These results are in contrast to the weak fidelity reported in a previous dental study [13], the only other published dental MI study assessing fidelity.

This assessment of fidelity, while presenting difficulties in community-based work, will aid our understanding of the intervention process and outcomes. As a consequence of our in-depth fidelity examination, other researchers and clinicians will have a more complete understanding of the implementation of this MI intervention and its potential replication.

## Abbreviations

HE: Health education; MI: Motivational Interviewing; WIC: Women, Infants and children centers.

## Competing interests

The authors declare that they have no competing interests.

## Authors’ contributions

PW conceptualized the fidelity study, carried out the training, and wrote initial drafts of final paper. PM edited and rewrote the initial and final drafts of the manuscript and contributed to the final manuscript. CAR contributed to the conceptualization, helped define the variables, and contributed to the manuscript. LAM had primary responsibility for the statistical analyses and contributed to the manuscript. GG managed the data, produced the figures, and contributed to the paper. CEH contributed to the conceptualization, helped define the variables, and contributed to the manuscript. DS and MS supervised the counselors in the field and contributed to the manuscript. AN served as a coder and contributed to the manuscript. All authors read and approved the final manuscript.

## Pre-publication history

The pre-publication history for this paper can be accessed here:

http://www.biomedcentral.com/1472-6831/14/15/prepub
